# Synthesis of Metallic Nanocrystals: From Noble Metals to Base Metals

**DOI:** 10.3390/ma12091497

**Published:** 2019-05-08

**Authors:** Liuyang Bai, Yuge Ouyang, Jun Song, Zhi Xu, Wenfu Liu, Jingyu Hu, Yinling Wang, Fangli Yuan

**Affiliations:** 1 Zhumadian Academy of Industry Innovation and Development, Huanghuai University, Zhumadian 463000, China; songjun@huanghuai.edu.cn (J.S.); chuangsheng0809@163.com (Z.X.); 2 State Key Laboratory of Multi-Phase Complex Systems, Institute of Process Engineering, Chinese Academy of Sciences, Beijing 100190, China; ygouyang@ipe.ac.cn (Y.O.); flyuan@ipe.ac.cn (F.Y.); 3 Department of Mechanical and Energy Engineering, Huanghuai University, Zhumadian 463000, China; liuwenfu@huanghuai.edu.cn (W.L.); hjy8868@huanghuai.edu.cn (J.H.)

**Keywords:** nanocrystals, noble metal, base metal, synthesis method

## Abstract

Metallic nanocrystals exhibit superior properties to their bulk counterparts because of the reduced sizes, diverse morphologies, and controllable exposed crystal facets. Therefore, the fabrication of metal nanocrystals and the adjustment of their properties for different applications have attracted wide attention. One of the typical examples is the fabrication of nanocrystals encased with high-index facets, and research on their magnified catalytic activities and selections. Great accomplishment has been achieved within the field of noble metals such as Pd, Pt, Ag, and Au. However, it remains challenging in the fabrication of base metal nanocrystals such as Ni, Cu, and Co with various structures, shapes, and sizes. In this paper, the synthesis of metal nanocrystals is reviewed. An introduction is briefly given to the metal nanocrystals and the importance of synthesis, and then commonly used synthesis methods for metallic nanocrystals are summarized, followed by specific examples of metal nanocrystals including noble metals, alloys, and base metals. The synthesis of base metal nanocrystals is far from satisfactory compared to the tremendous success achieved in noble metals. Afterwards, we present a discussion on specific synthesis methods suitable for base metals, including seed-mediated growth, ligand control, oriented attachment, chemical etching, and Oswald ripening, based on the comprehensive consideration of thermodynamics, kinetics, and physical restrictions. At the end, conclusions are drawn through the prospect of the future development direction.

## 1. Introduction

Nanomaterials are materials sized from 1 to 1000 nm in at least one dimension, and usually from 1 to 100 nm. Nanoparticles are one-dimension (1D) nanomaterials that may be as small as the atom and molecular in scale. There are two different forms of nanoparticles: amorphous or crystalline form, and nanocrystals are the crystalline form nanoparticles. This nano-particulate matter can be regarded as a unique material state to some degree, different from the solid, liquid, and gaseous states. For example, fullerenes and carbon nanotubes are materials in crystalline nanoparticle form, while graphite and diamond are of traditional crystalline solid forms [1]. Surface effects and quantum effects are the two primary properties of nanomaterials that make them show significantly different characteristics from bulk materials. Surface effect refers to the change in properties of nanomaterials caused by the sharp increase of the ratio of surface atoms to total atoms as the particle size decreases. The crystal field environment and binding energy of surface atoms are different from those of internal atoms. Surface atoms lack adjacent atoms and have many suspended bonds. They are unsaturated and easy to combine with other atoms to stabilize. Therefore, they show positive chemical and catalytic activity [2]. Chemical reactivity and the mechanical, optical, electrical, and magnetic properties of nanomaterials are all influenced by this surface effect. For example, Au particles of 10nm can absorb green light and show red color, and particles of 2–3 nm show considerable magnetic properties, which differ greatly from bulk Au materials. Furthermore, Au nanoparticles will become insulators as the size decreases to nanoscale. In the field of catalysis, although bulk Au has no catalytic activity when CO is oxidized to CO_2_, Au nanocrystals around 3.5 nm can catalyze the reaction and exhibits excellent catalytic activity [2]. Nanotechnology is the science and technology related to design, synthesis, and application of nanomaterials. Metallic nanomaterials, which contain the special properties of both nanomaterials and metal materials, has made great contributions to understanding the nano-world during the development of nanotechnology [3,4]. Due to their unique size and morphology, metal nanocrystals are different from conventional materials in thermal, optical, electrical, and magnetic aspects, making them the most dynamic materials in the field of research and development, and have great influence on economic and social development [5].

Metal catalysts exhibit unique physical and chemical properties, and have been widely used in industrial catalysis since the 20th century. In 1831, Phillips from British discovered that precious metal Pt could catalyze the oxidation reaction, producing sulfur trioxide from sulfur dioxide [6,7]. Then, in 1875, Jacob from German applied this work to industrial production, and established the first contact device for the production of fuming sulfuric acid, which opened a new era for the development of metal industrial catalysts [7,8]. As the pioneer catalyst, Pt remains the key component of many important reactions. Compared with traditional bulk metal, nanocatalysts exhibit higher catalytic conversion efficiency due to their higher specific surface area, thus reducing the amount of materials and production costs. Especially with the development of controllable synthesis of nanocrystals having complicated components, size, morphologies and exposure crystal plains, the applying of nanotechnology to the research of catalytic chemistry has become one of the most available approaches in this field. On the other hand, the research methods in homogeneous catalysis based on single center of complexes have become mature. The sub-nanometer scale, as the connection of atomic scale and nanoscale, homogeneous catalysis and heterogeneous catalysis, brings about a new possibility of exploring the catalytic process and developing highly efficient catalyst. In 1926, Murray Raney prepared porous nano-alloys by etching NiAl alloys, which was the famous Raney Ni [9]. This porous structure greatly increased the surface area and the catalytic activity for hydrogenation of vegetable oil. However, researchers had not paid enough attention to the chemical synthesis process of Raney Ni at that time, nor had they realized that superior metal nanocrystals having controlled size, varied morphology and adjustable composition would play such important role within the chemical catalysis field, which was worthy of thoroughly exploring. Until the 1990s, nanotechnology began to make significant breakthroughs based on liquid-phase chemical synthesis. Various kinds of nanocatalytic materials have been generated using this simple and effective synthesis route. The heterogeneous catalytic reaction of metal catalysts usually begins with reactants being adsorbed onto the catalysts’ surface, followed by the breaking or reconstruction of some chemical bonds between them, resulting in products. If the reaction continues on the surface, the products must be desorbed continuously away from the surface of catalysts. It was found that the size, morphology and structure of the catalyst would affect the electronic state of the catalyst surface, thus determining the catalytic efficiency [8].

The size change will have effect on catalytic performance for a reaction. When the nanocrystal size is reduced, the surface area of active components will increase and the utilization efficiency of catalytic active components will be improved. At the same time, with the decrease of the coordination number of surface atoms, the hanging bond will increase, the surface energy of nanocrystals will increase, and the catalytic performance will be enhanced accordingly [10]. Recent studies based on theoretical and experimental research results have shown that sub-nanoscale clusters exhibit higher conversion, selectivity, and stability than nanocrystals in some reactions. For example, Zhang et al. prepared Ir/Fe(OH)_x_ catalysts by co-precipitation method, the activity of which intensified by more than one order of the magnitude and had higher CO_2_ poisoning resistance compared to the best result of reported catalysts (Au/Fe_2_O_3_-WGC). Furthermore, the application of mono-atom in catalysis has been gradually reported. For example, Lee et al. observed that the mono-atomic catalyst Pd/meso-Al_2_O_3_ exhibited the highest activity in alcohol oxidation, and the catalytic activity decreased with the increase of size [11]. It should be pointed out that the excellent catalytic performance of small size is also influenced by the reaction system. There exists no linear relationship between catalytic performance and particle size. For a specific reaction, too strong or too weak adsorption of intermediates on catalysts will change the catalytic performance of materials. Therefore, it is necessary to find the optimal binding energy by changing the size. Shao et al. compared the redox activity of Pt with different sizes of 1–5 nm and found that Pt had the highest activity with the size of 2.2 nm, and the specific surface area activity increased slightly when the size was larger than 2.2 nm. The theoretical calculation results show that the particle size of 2.2 nm has the lowest oxygen adsorption energy, and the adsorption energy below or above the particle size is higher, which is not conducive to the chemical reaction [12,13].

The morphology change will also affect the catalytic performance for a reaction. The surface charge distribution of metal nanoparticles follows the tip effect of gathering electricity. The larger the surface curvature and the more prominent it is in other parts, the more the charge distribution is. Sargent et al. introduced this common physical concept into electrocatalytic reaction for the first time and found that the carbon dioxide conversion efficiency of acicular Au nanocrystals was much higher than that of Au rods and Au spheres with slightly smaller surface curvature at low voltage [14]. Theoretical simulation shows that the smaller the tip curvature of needle-like Au nanocrystals, the higher the electron density, and the stronger the electric field around them. The strong electric field will accumulate a large number of potassium ions and enrich carbon dioxide, which is conducive to carbon dioxide activation. Similarly, in photocatalytic reactions, prominent samples can also aggregate light. Xiong et al. prepared a concave Pd nanocrystals. The sharp edges of this structure have super-strong light-gathering ability, which can produce local high temperature and promote the hydrogenation reaction at the edges. The catalytic efficiency of the Pd nanocrystals can reach 70 degrees under room temperature spectral irradiation [15]. Li et al. studied the silver nanoparticles on their catalytic activity in the oxidation reaction of styrene, and found that the crystal shape had great effect on the catalytic activity. The reaction rate of nanocubes was 15 times of nanoplates and five times of nanospheres [16].

The electronic structures of different crystal surfaces will also affect the catalytic performance for a reaction. The construction of nanocrystals with bare surface is an effective means to control the electronic structure of materials. The arrangement of atoms on the {111} crystal plane is the densest hexagonal packing with lower surface energy, while the {100} crystal plane is a lattice array with higher surface energy. The different arrangement of surface atoms leads to the difference of coordination number of surface atoms, which affects the surface crystal energy of the catalyst. In face-centered cube metals, the order of surface energy of three groups of common low exponential crystalline planes is {111} < {100} < {110} [17,18]. The difference will inevitably affect the adsorptive capacity of the reactants, which will further lead to different catalytic activity or selectivity. In addition, the coordination number of these atoms is lower due to the existence of numerous edges, angles and step atoms, and they tend to show higher activity in catalytic reactions. Zhang et al. prepared Pt polygonal rod structure with surface exposed {211} crystal plane and Pt octagonal rod structure with surface exposed {411} crystal plane and used them in the electrocatalytic oxidation of ethanol. The order of catalytic activity of Pt nanocrystals on different crystal surfaces is {411}> {211} > {100} [19]. In Li’s work mentioned above, difference in catalytic activities shown by different shape of silver nanoparticles were not caused by surface area change and the varied atom fraction of edges or corners, but instead were related to the exposed crystal surface. The nanospheres consisted of both the {111} and the {100} facets, the nanoplates were mainly composed of the {111} facets, which is the most stable facets, and the nanocubes predominantly consisted of less-stable {100} facets [16]. Moreover, it was predicted based on computer simulations that high surface energy would make it easier when ethylene or oxygen were adsorbed and subsequently activated onto crystal surfaces [20]. Based on the above results, it was concluded that when silver nanoparticles are applied as catalysts, the order of the activity was that {111} < {100} < {110}.

Nanocrystal synthesis is the basis of performance research and application, so synthesis plays the core role in nanotechnology. There are many studies on synthesis of metallic nanocrystals and significant process has been made almost every year. The present review covers the most important methods used in metal nanocrystal synthesis. Specific examples and strategies in synthesis of metallic nanocrystals are introduced, and the metals include both precious metals Pt, Pd, Ag, Au, base metals such as Cu, Ni, Co, and their alloy nanocrystals. Successful synthesis of noble metal and alloy nanocrystals are summarized, followed by the challenges in base metallic nanocrystal synthesis and the perspectives on future directions. Our intention is to make use of the successful experiences of noble metal to promote the synthesis of base metal nanocrystals.

## 2. Methods for Metallic Nanocrystal Synthesis

### 2.1. Typical Redox Process

Synthesis of metallic nanocrystals are typically related to a redox reaction process, in which salt precursor is chemically reduced by reductants. Electron transfer would be involved between chemical species due to the different reduction potentials of the two raw reagents [21]. Generally, the process of electron transfer is described by two half reactions. The oxidation half reaction is Equation (1), the reduction half reaction is Equation (2), and the total redox reaction can be given as Equation (3), in which reductant species is labelled as *R_r_*, oxidized species is labelled as *R_o_*, metal ion is labelled as *M*^*n*+^, and metal atom is labelled as *M*^0^ [22]. *M*^*n*+^ would be reduced to *M*^0^ when it receives electrons from *R_r_*, and *R_r_* would be oxidized to *R_o_* upon giving electrons to *M*^*n*+^.

(1) $$R_{r}\to R_{o}+ne^{-}$$

(2) $$M^{n+}+ne^{-}\to M^{0}$$

(3) $$R_{r}+M^{n+}\to R_{o}+M^{0}$$

Table 1 and Table 2 summarize the oxidation potentials of various metal salts and reductants which are mostly chosen for the fabrication of metallic nanocrystals [23]. Appropriate reductant can be selected according to oxidation potentials, and the rate of the redox reaction, which always has great effect on the nucleation and crystal growth, can be appropriately controlled and adjusted accordingly. Taking the synthesis of Ni particles via hydrazine reduction of nickel chloride hexahydrate in basic aqueous solution for example, the reaction can be described as follows:

(4) $$N_{2}H_{4}+4OH^{-}\to N_{2}+4H_{2}O+4e^{-}$$

(5) $$Ni^{2+}+2e^{-}\to \mathrm{Ni}$$

(6) $$N_{2}H_{4}+4OH^{-}+2Ni^{2+}\to N_{2}+4H_{2}O+2\mathrm{Ni}$$

(7) $$\Delta E=\Delta E_{o}+\Delta E_{r}=-0.25V+1.16V=0.91V$$

Oxidation half reaction is as Equation (4), reduction half reaction is as Equation (5), and the total redox reaction is as Equation (6). Redox reactions are thermodynamically favorable with positive ∆*E* value, so the hydrazine reduction of the Ni^2+^ occurs spontaneously in basic condition. However, results are quite different when the reaction system was in acidic aqueous solution. The calculation of ∆*E* is given below:

(8) $$\Delta E=\Delta E_{o}+\Delta E_{r}=-0.25V+0.23V=-0.02V$$

Redox reactions are thermodynamically unfavorable with negative ∆E value, so the hydrazine reduction of the Ni^2+^ will not occur spontaneously in acidic condition, which means that Ni particle can be synthesized via hydrazine reduction of nickel chloride hexahydrate only when the pH value was high enough.

### 2.2. Polyol Method

Polyol method is recognized as a facile route to metallic nanoparticles. It was first reported by Fiévet and co-workers in 1989 and then developed by various researchers to prepare diverse metallic nanocrystals such as Au, Ag, Cu, Fe, Co, Ni, Pt, Pd, Ru and Ir, and also alloys such as CoNi [24,25]. As a matter of fact, polyol method plays roles so important in the synthesis of metallic nanocrystals that we need to introduce it in a separate section. The mechanism of the polyol reduction varies according to the reaction temperature. Equations (9)–(13) show the different reduction pathway dependent on temperature.

(9) $${\mathrm{HOCH}}_{2}{\mathrm{CH}}_{2}\mathrm{OH}\to {\mathrm{CH}}_{3}\mathrm{CHO}+H_{2}O$$

(10) $$2{\mathrm{nCH}}_{3}\mathrm{CHO}+2M^{n+}\to 2M^{0}+{\mathrm{nCH}}_{3}{\mathrm{COCOCH}}_{3}+2{\mathrm{nH}}^{+}$$

(11) $$2{\mathrm{HOCH}}_{2}H_{2}\mathrm{OH}+O_{2}\to 2{\mathrm{HOCH}}_{2}\mathrm{CHO}+2H_{2}O$$

(12) $${\mathrm{nHOCH}}_{2}\mathrm{CHO}+2M^{n+}\to 2M^{0}+\mathrm{nHOCCHO}+2{\mathrm{nH}}^{+}$$

(13) $${\mathrm{nHOCH}}_{2}{\mathrm{CH}}_{2}\mathrm{OH}+2M^{n+}\to 2M^{0}+{\mathrm{nHOCH}}_{2}\mathrm{CHO}+2{\mathrm{nH}}^{+}$$

Ethylene glycol is dehydrated to generate acetaldehyde at the temperature above 160 °C as shown in Equation (9), and the acetaldehyde plays the real role of reductant to reduce metal ions as shown in Equation (10) [26]. At a temperature between 140–160 °C, ethylene glycol would reaction with oxygen and produce glycolaldehyde as shown in Equation (11), and glycolaldehyde is responsible for the metal reduction as shown in Equation (12). At a temperature below 140 °C, ethylene glycol is responsible for metal reduction by itself, as shown in Equation (13) [21].

Polyol is expensive as the reductants compared to hydrazine and hydrogen. However, this method exhibits lots of irreplaceable advantages which makes it popular and practical. Firstly, polyols can dissolve different precursor including many salts; Secondly, their relatively high boiling points provide them unique temperature-dependent reducing power, which helps control the nucleation stage and crystal growth process by regulating of temperature in a large range. Additionally, some reactive metals are difficult to reduce, such as Ni, Co, Cd, and Bi, can also be fabricated by decomposing their precursors in the such high-boiling-point solvents [27,28]. Furthermore, vapor pressure of ethylene glycol is low due to its high boiling point, which makes it more secure in the solvothermal synthesis using autoclaves.

The experimental setup for polyol synthesis is also very simple and easily available. Xia and co-workers have provided their typical synthesis procedure for Ag nanocrystals. AgNO_3_ and surfactant PVP (Polyvinylpyrrolidone) solution were made ahead of time by dissolving them into ethylene glycol, respectively. Another amount of ethylene glycol as mother liquid was heated in the three-necked flask at 160 °C for 1 h. Then the AgNO_3_ solution and the PVP solution were shot simultaneously into the mother reaction solution [29]. The elemental silver could form at proper rate because the power of reducing ability of ethylene glycol is highly dependent on the temperature of reaction [30]. Nucleation and crystal growth process will be detailed in the sections below.

### 2.3. Crystal Growth

**Figure 1:**
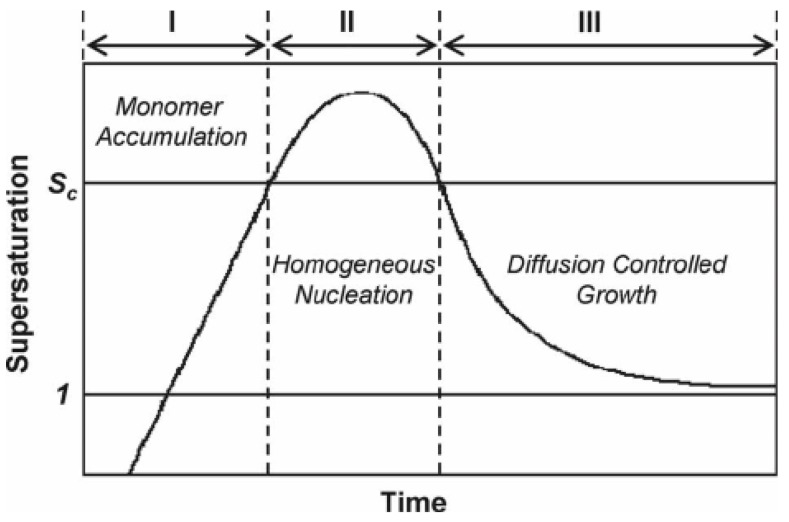
Nucleation and crystal growth. Reproduced with permission [31]. Copyright 1950, American Chemical Society.

According to classical thermodynamic theory, one can divides the crystallization process of nanocrystals into two parts: the nucleation stage and the crystal growth stage, both of which are very complex physical and chemical process, and are affected by many parameters in the chemical reaction. In 1950, Lamer proposed a theoretical model for typical nucleation and crystal growth in solution, in which the nucleation and growth was mainly divided into three stages: monomer accumulation, homogeneous nucleation, and diffusion-controlled growth. The illustration is shown in Figure 1.

Firstly, the precursor is reduced or decomposes upon heating in solution and produces a large number of atoms, and then the atoms forms stable nuclei when their concentration in solution exceeds a certain supersaturation threshold. Secondly, the nucleation accelerates and leads to the decrease of metal atom concentration in solution, and no new nuclei will generate in the solution when the concentration is lower than the supersaturation value. Finally, the atoms produced by precursor decomposition diffuse to the surface of the nucleus, and the nucleus grows up until the atoms on the nanocrystal surface reach the diffusion equilibrium with the atom concentration in the solution, and then the reaction terminates [13,31]. Because of the complexity of the reaction process and the limitation of the characterization instruments, it remains challenging to observe directly the nucleation or crystal growth process in the experimental process. However, thanks to the development in characterization instruments and experimental techniques, especially the in-situ characterizations, it gradually becomes possible to further observe directly the nucleation or crystal growth [32,33,34]. For example, Wei’s group used in situ ultraviolet-visible absorption spectroscopy and in situ synchrotron radiation technology for comparing the influence of different nucleation paths on the final shape of nanocrystals [35]. Cl_3_Pt-Cl_3_Pt with dimer intermediate state was obtained in weak ethylene glycol reductant, and zero-valent Pt atom was obtained in strong reductant citric acid. In the subsequent reaction, different intermediate products were finally reduced to different morphologies. For example, dimer Cl_3_Pt-Cl_3_Pt grows into nanowire structure, and zero-valent Pt atoms gather together to form nanoparticles. In addition, the in-situ observation technique can be used to examine the surface information and also the electronic structure in clusters, atomic occupancy during the diffusion and corrosion of nanocrystals and structural changes during the reaction, and to understand the whole reaction process from the atomic level [36]. The development of in situ spectroscopy is of great significance for understanding nucleation and crystal growth.

Homogeneous nucleation and crystal growth are usually realized by thermal injection and temperature-rise reduction [13]. However, most of the complex structures are difficult to obtain by homogeneous nucleation. Seed-mediated method is another important method to construct nanocrystal structures, also known as post-synthesis method [37]. This method originated from Czochralski (Cz) method that were commonly used for fabrication of bulk single crystals [38]. Then, Murphy and co-workers demonstrated that Cz method was also suitable for Au nanorod fabrication using its colloidal nanoparticles as seeds. It was demonstrated by Xia’s group that silver pentagonal nanowires could be fabricated using Pt colloidal nanoparticles as mediated seeds [39,40,41]. Seed-mediated growth separates crystal growth from homogeneous nucleation, and provides an effective way to avoid homogeneous nucleation through direct deposition of atoms on prefabricated seeds with definite characteristics [37]. It further modifies and constructs the pre-synthesized substrate material, and achieves the structure that cannot obtain under general conditions. Seed-mediated method mainly includes heteroepitaxy growth, seed diffusion, metal substitution reaction and coordination corrosion. Heteroepitaxial growth means that different metal precursors nucleate and grow on the pre-synthesized seeds, resulting in composite nanostructures different from seed structures. Seed diffusion method refers to the process in which an external precursor does not nucleate on its surface when it contacts the seed, but diffuses into the lattice of the seed to form alloy structure with the seed. Galvanic replacement occurs only when the reduction potential of seed element is less than external element, in which seed particles are partially oxidized and dissolved in solution, and the metallic precursors are reduced to grow onto the seed. Coordination-etching refers to obtaining frame or concave structure in bimetallic or polymetallic nanocrystals by using different chelating ability between different components and ligands [13].

The use of ligand is another way to regulate the synthesis process and grain growth. On one hand, ligand can bind to metal cations to reduce the redox potential of metal cations, which makes the reduction more difficult and slower. On the other hand, ligand and its decomposition products can also be used as surfactants to stabilize some crystal planes and lower their growth. The stabilized facets would remain as the exposed crystal facets of the synthesized nanocrystal product. When the metal complex has a high reduction potential, the reducing will be a fast process, in which the high-energy facets have more chance to be retained and thermodynamically unstable structures would exist [42,43].

Corrosion has also been developed as a method to fabricate nanoparticles with controllable size and shape [44,45]. Xia combined corrosive pitting and etching together and fabricate Pd nanoboxes or nanocages from nanocubes via a one-pot method with no exotic templates [46]. Another example is the selective removal of twinned seeds by O_2_/Cl corrosion, leaving single-crystal truncated octahedra as the main product. On the contrary, C_6_H_8_O_6_ or C_6_H_8_O_7_^2−^ can block the O_2_/Cl^−^ corrosion by way of binding to crystal facet {111}, and the use of C_6_H_8_O_6_ or C_6_H_8_O_7_^2−^ facilitates the growth of icosahedra and decahedra [47].

## 3. Synthesis of Noble Metal Nanocrystals

### 3.1. Au Nanocrystals

Au nanocrystals with low-index surface including {100}, {111}, and {110} exposed are relatively easy to be obtained. Both single-crystal and twinned-crystal structures were synthesized by different scientists and the morphology includes cubes, octahedra, cuboctahedra, tetrahedra, rhombic dodecahedra, rods, prisms, decahedra, icosahedra, and wires. Recently, progress has been also made in the fabrication of nanocrystal Au enclosed with high-index surface, such as {221}, {037}, {310}, {720}, and {321} facets [48].

**Figure 2:**
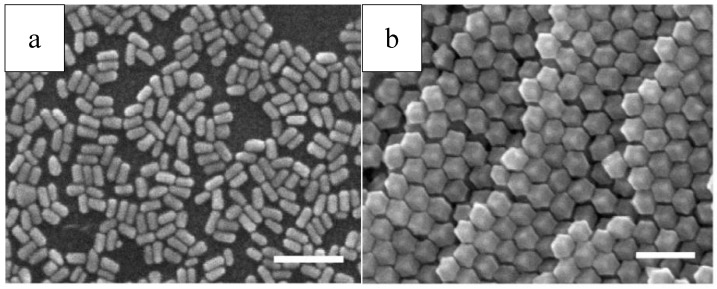

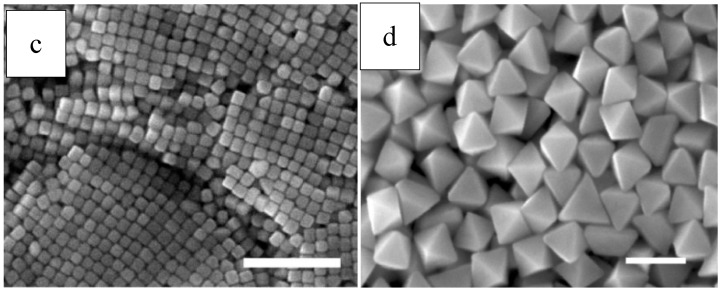
Single-crystalline Au nanocrystals with different shape (**a**). nanorods; (**b**). rhombic dodecahedra; (**c**). cubic nanocrystals; (**d**). octahedral nanocrystals). Reproduced with permission [51]. Copyright 2009, American Chemical Society.

Single-crystals: San et al. reported a chemical reduction route in solution that could produce a series of Au nanoarchitectures such as cubes, rods, stars, triangle, rectangle, hexagon like, and branched particles. The high-yield synthesis was conducted at normal temperature using CTAB for a surfactant [49]. Yang and co-workers reported a method for preparation of uniform Au isotropic nanostructures of cubes, octahedra, tetrahedra, and icosahedra in high yield using the improved polyol process, using ethylene glycol as both reducing agent and solvent [45]. Seo et al. have prepared Au polyhedral nanocrystals also via an improved polyol process, in which gold precursors were rapidly reduced from the refluxing 1,5-pentanediol. A number of Au nanocrystals such as truncated octahedron, cube, octahedron, higher polygon, and cuboctahedron were obtained by changing the concentration of silver nitrate gradually [50]. Niu reported a facile seed-mediated fabrication route to single-crystalline Au nanocrystals including nanocube, rhombic dodecahedron, and octahedron, as shown in Figure 2. To study the mechanisms for the Au nanosized single-crystals in varying shapes, kinetics and surface-energy were also taken into consideration. It was found that the surfactant CPC could change the Au surface energy, and the order is {100} > {110} > {111}. Accordingly, the provided growth condition kinetically leads to the structures less stable instead of thermodynamically favored ones [51]. Huang reported on the preparation of Au nanocrystals in the aqueous solution, and the shape of the structures was systematically controlled as cube, truncated cube, rhombic dodecahedron, and trioctahedron. It was found that the application of both CTAC and NaBr could control the bromide concentration, which was the critical parameter for the nanocube formation. Nanocrystal morphology could be precisely controlled by varying the amount of ascorbic acid that added into the reaction solution [52]. Jana et al. synthesized nanorods with an aspect ratio high enough without using any mesoporous templates. Instead, an improved seed-mediated growth method using water-based micelles as templates contributed to the one-dimensional growth. The aspect ratio of Au nanorods could be controlled by varying the growth parameters [53]. Zheng has synthesized nanospherical gold single crystals with controllable size ranging 5–150 nm successfully by using seed-mediated method repeatedly. Firstly, synthesis of gold nanospheres with tunable size of 5–16 nm by changing the number of Au clusters capped by CTAB. Secondly, synthesis of gold nanospheres with tunable size of 15–80 nm using Au nanospheres obtained in first step as a second-round seeds. The strategy was repeated in the next step using the Au nanospheres obtained in the previous step, by which they synthesized nanospheres as big as 70–150 nm. It was found in the experiments that the method of adding precursor, the concentration of reductant, and the existence of halide anions were critical parameters for high-yield production of Au nanospheres with single-crystal structure and with uniform particle size that can be controlled ranging 5–150 nm [48]. 

Twinned crystals: Seo et al. reported a one-pot method in which diethylene glycol was used as solvent and reductant, and HAuCl_4_ was the precursor and PVP was the surfactant. Under an identical reaction condition, both single-crystal and twinned-crystal structures were synthesized successfully. Polyhedral shapes could be altered by changing the concentration of PVP. When the concentration was high, the seeds were stabilized by PVP sufficiently and the decahedral structure would be retained during crystal growth. When the concentration was low, icosahedral structure existed so that the crystals’ surface energy could be reduced. Therefore, octahedral and truncated tetrahedral single-crystals were produced at low concentration of PVP, because that low concentration of PVP lead to the decrease of particle surface blocking by PVP, which further facilitated oxidative etching from Cl^−^/O_2_ [54]. Li et al. also prepared truncated bipyramid with singly twinned structure when HAuCl_4_ was reduced by N-vinyl pyrrolidone using water as solvent. However, single-crystal Au octahedron could also be obtained with the assistance of CTAC when the amount was suitable. Mechanistic studies explained that the formation process of Au truncated bipyramid and octahedron in identical reaction condition was related to oxidative etching. Oxygen originated from air, and a Cl ligand was brought about by the reactants, which would act as a forceful etchant during both nucleation and crystal growth stages [55]. Liz-Marzán et al. synthesized a new class of extremely regular gold nanocrystals. Decahedra in high yield has been achieved through ultrasound-induced reduction of HAuCl_4_ using PVP as a stabilizing polymer. Pre-synthesized gold seeds were applied, and the dimensions could be controlled through the ratio between the amount of seed and the HAuCl_4_ concentration [56].

**Figure 3:**
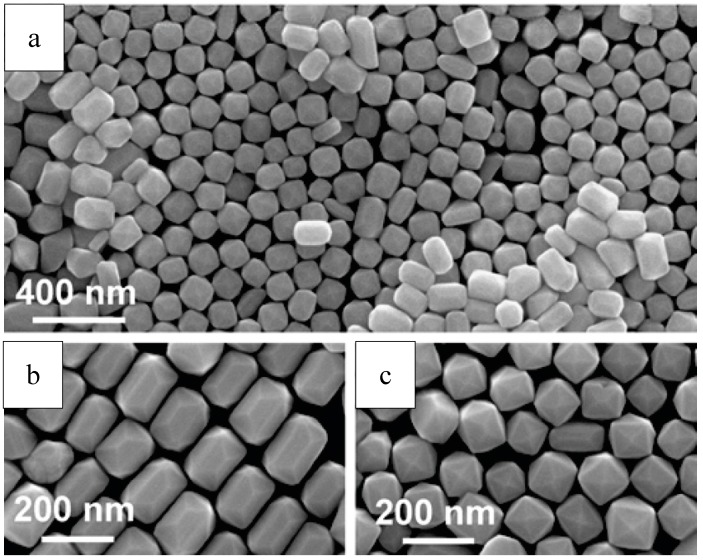
Large-area SEM image of the tetrahexahedral Au nanocrystals deposited on a Si substrate (**a**) and their side and end facets (**b**,**c**). Reproduced with permission [61]. Copyright 2009, American Chemical Society.

High-index-facet crystals: Zheng et al. reported the successful high-yield synthesis of Au nanorice with {611} exposed facets. AuI-TEG complex was used as precursor and Ag^+^ ions presented as mediator. Penta-twinned seeds formed at the beginning stage, and then further grew into nanorice [57]. The catalytic activity of this novel crystal structure was higher than conventional Au multiply twinned crystals exposing {111} facets with similar particle size. Furthermore, the synthesized nanorice was stable when it was used in the reaction of CO oxidation. Millstone et al. provided a method for synthesis of uniform Au nanoprisms. Small Au seeds were prepared first, and then a three-step growth was conducted subsequently onto the seeds. The Au precursor was HAuCl_4_, the capping agent was CTAB, the reducing agents was ascorbic acid, the solvent was water, and NaOH was used as pH value regulator [58]. Zhang discovered novel Au concave nanocubes exposing high-index facets of {720}. The synthetic method was developed on the basis of seed-mediated process, in which Cl^−^ counterion brought about by the surfactant had great effects on the formation of concave nanocrystals [59]. Ma reported large quantity synthesis of novel Au trioctahedral nanocrystals exposing high-index facets including {221}. Experiments were carried out under room temperature. Typically, CTAC aqueous solution was appended to an HAuCl_4_ solution, and HAuCl_4_ was reduced by AA with the existence of CTAC at ambient temperature [60]. Ming et al. synthesized elongated THH Au single crystals exposing high-index facets of {037} facets via seed-mediated growth. Figure 3. shows SEM image of the tetrahexahedral Au nanocrystals deposited on a Si substrate [61].

### 3.2. Ag Nanocrystals

Synthesis of Ag nanocrystals also mainly focuses on the shape control and exposed crystal facet control. We will review this part of work according to the applied methods in order to avoid duplication with the previous section.

**Figure 4:**
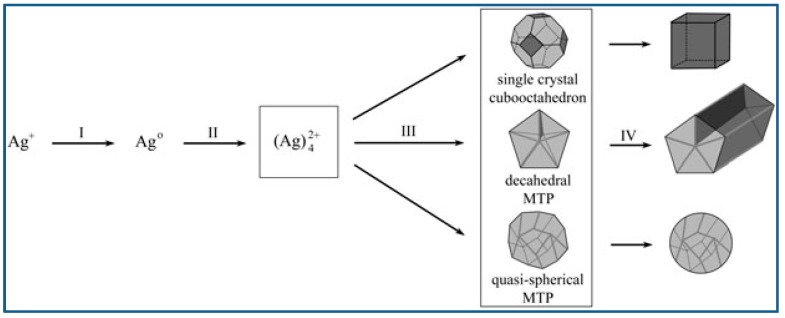
Schematic illustration of reduction of Ag^+^ ions by ethylene glycol. Reproduced with permission [29]. Copyright 2005, Wiley-VCH.

Polyol method has already been developed successfully to synthesize well-defined nanocrystals. Xia and co-workers successfully produced different Ag nanocrystals such as cube, rod/wire, and summarized three typical nanostructures of them produced via the route of polyol process mediated by PVP, as shown in Figure 4 [29].

A typical synthesis procedure was presented in Section 2.2, and the formation of Ag nanocrystals can be described as follows: First, Ag^+^ from the solution was reduced using ethylene glycol and elemental silver generated at a moderate rate [30]. Second, Nuclei appeared when the elemental Ag concentration rose to the critical value [62]. Third, Ag nanoparticles were generated in roughly spherical profile, which can be observed that when the PVP and AgNO_3_ were added the reaction solution became yellow [63]. Forth, the subsequent generated silver atoms would not aggregate to clusters again; instead, they diffused to the nuclei surface generated in former step, and occupied the active sites of the surface and formed chemical bonds with the atoms around them [29].

The shape of the resulting nanocrystals can be controlled by changing mole ratio of PVP/AgNO_3_. Because the surface coverage of PVP and the thickness of coating could be modified with the variation of PVP/AgNO_3_ mole ratio, which further changed the growth resistance of the facets, and lead to the production of silver nanocrystals with various morphology [29]. Nanocubes were obtained with a high concentration of AgNO_3_ at 0.125–0.25M and a low mole ratio PVP/AgNO_3_ of ~1.5. Nanowires were produced when the concentration of AgNO_3_ were reduced and the ratio PVP/AgNO_3_ were kept constant. On one hand, low precursor concentration would lead to sufficiently low level of the chemical potential, in which multiple-twined nanocrystals instead of single-crystal nanocrystals would form. On the other hand, Ag atoms prefer to crystallize onto the twinned defect on the MTP’s (Multiple-Twined Particles) surface because the defect site possess highest energy. Decahedra elongate uniaxially into pentagonal nanorods, the sides of which are confined by facets of {100}. Furthermore, due to the stronger interacts between PVP and {100} facets than {111}, the side facets of nanorods are passivated sufficiently by PVP, while the end facets keep reactive and ready to receive more silver atoms. Therefore, the nanorods can further grow long and long into nanowires [18,29].

Other kinds of capping agents can also be used as shape-control reagents. For example, when sodium citrate was applied as capping agents, the Ag {111} facet instead of {100} facet was promoted, leading to the generation of nanobelts or triangular nanoplates that exposed {111} facets [64]. Yam and co-workers developed a system that can synthesize Ag nanocrystals with a variety of shape by the addition of glucose and CTAB into silver mirror solution of [Ag(NH_3_)_2_]OH. AgBr was the main existing form of Ag(I), and the [Ag(NH_3_)_2_]^+^/Ag reduction potential showed a negative shift [65,66]. Therefore, the reaction with the existence of CTAB can be controlled in a relatively large temperature range compared to the traditional Ag mirror reactions. Different nanocrystals such as nanocube, triangle, nanorod, and nanowire, were obtained at different mole ratio of CTAB/[Ag(NH_3_)_2_]OH [67].

Ag nanocrystals were also produced in other solutions besides ethylene glycol. Sun reported an Ag triangular nanoplate and nanobelt synthesis method, in which AgNO_3_ was reduced by NaBH_4_ with the existence of sodium citrate or PVP in aqueous solution. The reaction was refluxed at ambient temperature and atmosphere for 10 h. The products were composed of small Ag nanospheres at the beginning, and then the nanospheres gradually transformed into triangular nanoplates or nanobelts by way of Ostwald ripening. The final products consisted of both nanoplates and nanobelts with a ratio of about 95/5. The triangular faces of the nanoplates were composed of {111} planes and the single-crystal nanobelts were along the [101] axis [64,68]. Li and co-workers reported preparation of silver nanoplate structures in a DMF solution. The synthesized Ag single-crystal nanoplates with truncated triangular shape has {111} facets as their exposed planes. The concentration of AgNO_3_, the reaction temperature, and the surfactant/AgNO_3_ ratio are the three dominate parameters that can affect the shape and structure of the Ag nanocrystals [16].

### 3.3. Pd Nanocrystals

Pd nanocrystals with various morphologies has also been successfully fabricated by different synthesis methods, among which polyol method is still the mostly applied route. The synthesized nanocrystals so far include nanocube, nanobar, nanorod, cuboctahedron, etc. [69,70,71]. There are so many reported literatures that we can only provide a few typical specific works here and present some of the most excellent results.

**Figure 5:**
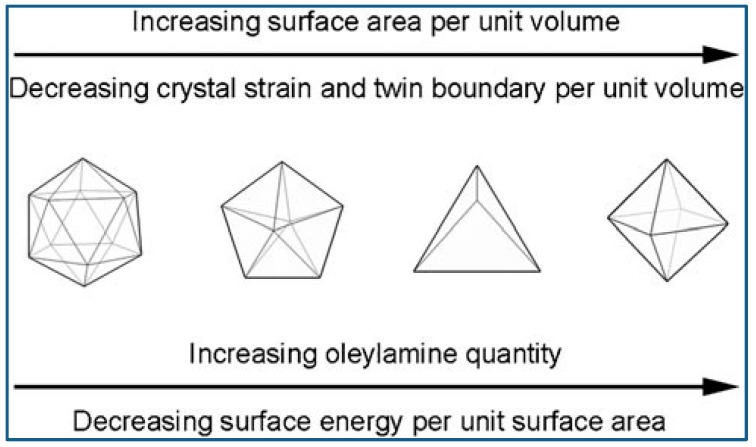
Schematic illustration of shape evolution mediated by OAm. Reproduced with permission [73]. Copyright 2009, American Chemical Society.

Niu et al. reported an OAm-based reaction for shape-controlled synthesis of Pd nanocrystals, and deca-like, icosa-like, octa-like, triangular platelike, and tetrahedral shaped nanocrystals were synthesized selectively by changing the amount of OAm. Typically, a certain amount of [Pd(acac)_2_], formaldehyde, toluene, and OAm were mixed and stirred at ambient temperature and atmosphere, followed by heated at 100 °C for 8 h in autoclave, and then slowly cooled to room temperature. Nanocrystals were formed via three steps: intermediates generation, nucleation formation, and crystal growth. The intermediates generation facilitates the manipulation of the reduction kinetics. The separation between nucleation and crystal growth provides precise control over the morphology and structure. OAm played very important roles during the shape evolution of Pd nanocrystals. The surface energy of the nanocrystals was dramatically reduced with the amount of OAm increased, because more surfaces were covered by surfactant. Therefore, multiple-twinned nanocrystals with low ratio of surface-to-volume evolved into single nanocrystals with high ratio of surface-to-volume [72]. To get better understanding of the Pd nanocrystal’s evolution in morphology, researchers employed a correlative thermodynamic model as a qualitative analysis means. Figure 5 shows the illustration of OAm mediated nanocrystal shape evolution [73].

**Figure 6:**
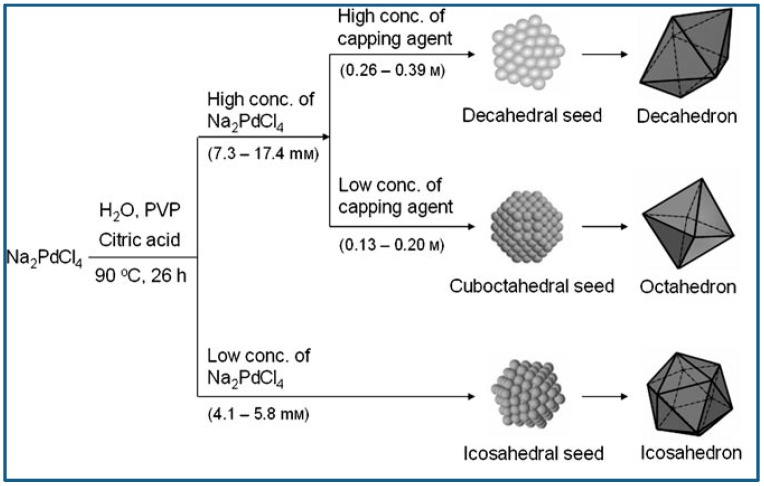
Reaction pathways leading to structure controlled Pd nanocrystals. Reproduced with permission [74]. Copyright 2007, Wiley-VCH.

Lim et al. reported controlled synthesis of Pd nanocrystals of various shape via reduction of Pd salts from their aqueous solution using citric acid as reducing agent. Typically, Na_2_PdCl_4_, citric acid, and PVP were dissolved in distilled water, and the aqueous solution was heated to 90 °C and kept at that temperature for 26 h. Na_2_PdCl_4_ was the metal precursor, citric acid served as not only reducing agent but also capping agent, and PVP was used as stabilizer. It was demonstrated citric acid was conducive to the generation of octahedral, icosahedral or decahedral structures enclosed by {111} surfaces, and the Pd nanocrystal shape varied dependent on the Pd precursor concentration and citric acid concentration. Icosahedral seeds can retain the small state for long time at low concentration of precursor, because the generation of Pd atoms and the diffusion of generated Pd to the icosahedral seeds was the slow. Therefore, icosahedra would predominate the final products when the precursor concentration was low and reaction and crystallization were slow. With the precursor concentration increased, however, Pd atoms generated fast, and decahedron and cuboctahedron would predominate the seeds due to their grown particle size. Figure 6 presents the reaction pathways leading to structure controlled Pd nanocrystals [74].

**Figure 7:**
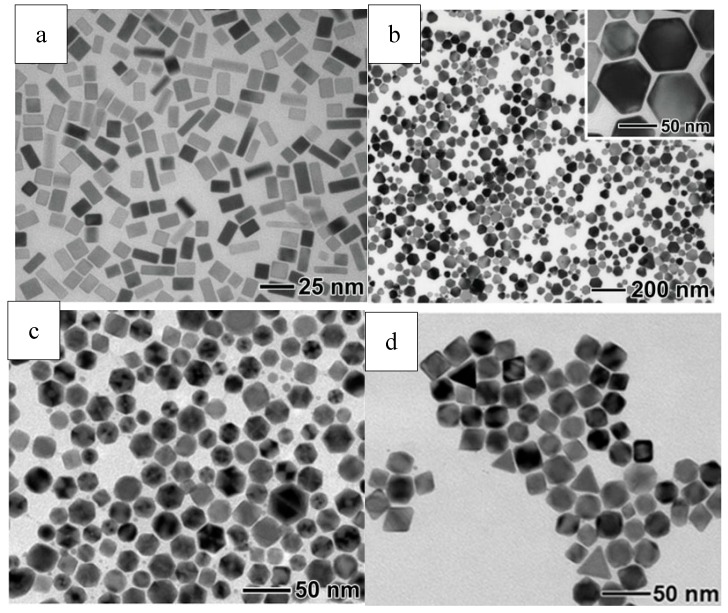
Typical Pd nanocrystals of different morphology (**a**). nanobars; (**b**). hexagonal and triangular Pd nanoplates; (**c**). icosahedrons; (**d**). octahedrons). Reproduced with permission [47]. Copyright 2009, Wiley-VCH.

Twin structure and crystal plane of seeds are most important factors determining the nanocrystal structure. Depending on the optimization of a series of parameters including basic reduction kinetics, traditional surface capping, and novel oxidative etching, Lim and co-workers have demonstrated the feasibility of producing palladium nanocrystals of various shapes such as truncated octahedron, decahedron, and icosahedron, cube, rectangular shaped bar, and thin plate in hexagonal and triangular outline. Reduction kinetics could be controlled by reductants selection, which further helped manipulate twin structures during the nucleation stage. Reactions were controlled by thermodynamics with a fast reduction rate, and single crystals or multiple-twinned crystals existed as the main form of seeds. These seeds can be regulated with the assistance of oxidative etching. Firstly, several twin seeds can be gotten rid of selectively by O_2_/Cl etching, leaving merely truncated octahedra as the seeds. Secondly, citric acid ion protects the seeds from oxidation etching by binding with {111} facets strongly, and promotes the generation of icosahedral and decahedral seeds. Reactions were controlled by kinetics with a considerably slow reduction rate, and nanoplates that had defects on their surface existed as the main form of seeds. These seeds further grew into nanoplates of hexagonal or triangular shape, which does not coincide with common thermodynamic behavior. In general, capping agents, which can bind to certain crystal facets, played a profound role in Pd’s crystallization behavior. Because of the strong interaction between the capping agents and the specific planes, the free energy order of different crystal planes could be changed. This chemical adsorption or surface coverage provides a way to control the relative rates of different aspects growth, leading to the formation of palladium nanocrystals of various shape and structure [47]. Figure 7 shows typical Pd nanocrystals of different morphology.

Other noble metallic nanocrystals such as Pt, Rh, and Ru were also successfully synthesized in addition to Au, Ag, and Pd. One specific example will be provided for each metal here. Chen and his workers synthesized Pt nanocrystals with four different shape by manipulating reduction kinetics of a polyol process. Nitrogen atmosphere present for different periods of time during the iron-mediated reduction had great effects on the morphologies. Star-like nanocrystals were produced when the nitrogen was supplied immediately after the formation of Pt(II) species, and branched nanocrystals were produced when the nitrogen was supplied after the Pt(II) species had been left in air at the temperature of 110 °C for 10 h [75]. Zhang reported a polyol synthesis of Rh tripods and starfish-like nanocrystals that have five arms. Tripods dominated the products when the synthesis was conducted in an anaerobic environment as to inhibit oxidative etching. Starfish-like nanocrystals dominated the products when [{(CF_3_COO)_2_Rh}_2_] was used as precursor to replace Na_3_RhCl_6_ as to further inhibit oxidative etching completely. Because Cl^−^ ions did not exist during the reaction process, high-yield production of nanocrystals of five-fold twinned structures that had five arms could be realized [76,77]. Kusada et al. reported their finding on metallic Ru crystals that pure fcc nanocrystals can be fabricated. Because of the nanosize effect, fcc Ru was obtained at room temperature, which never exists within ruthenium phase diagram for its bulk counterpart. Crystal types of fcc and hcp can be controlled when different precursors were used and the uniform nanoparticle can be adjusted in the size range of 2–5.5 nm [78].

## 4. Synthesis of Multimetallic Nanocrystals

### 4.1. Significance of Multimetallic Nanocrystals

Multimetallic nanocrystals are composed of noble metals and/or base metals. Specifically performance tests, the new type of materials often shows better performance than their corresponding single metal counterparts because of the “synergistic effect” between the two of them [79,80,81,82]. In addition, the introduction of a second non-precious metal into precious metals can reduce the use of precious metals, thus reducing the cost of synthesis of nanocrystals per unit mass. This is of great significance for practical application [83]. For example, Pt is the most widely used catalyst with the best performance. However, its practical application is still limited by the scarce resources and the slow kinetic process of oxygen reduction [84,85,86,87]. An effective technique is to alloy Pt with some abundant and base metals. Compared with single Pt metal, alloy nanostructures with less Pt content usually exhibit excellent properties [88,89,90]. Therefore, a series of Pt-based electrodes with good activity, such as PtMn, PtFe, PtCo, PtNi, PtCu and PtZn, were successfully prepared. Yang and his colleagues have reported a Pt_3_Ni nano-framework catalyst. The specific mass activity is 36 times that of commercial Pt/C, and the specific area activity is 22 times. [91]. Recently, Huang’s Group reported that Mo-doped Pt_3_Ni/C catalyst, the specific mass activity of which was 6.98 A/mg, and the specific area activity of which was 10.3 mA/cm^2^, 73 and 81 times that of commercial Pt/C, respectively [83,92].

### 4.2. Synthesis Methods of Multimetallic Nanocrystals

One-step method refers to the formation and compounding of two or more components to form composite nanocrystals, including wet chemical method, hydrothermal method, solvothermal method, etc. [93,94,95]. For example, Huang and co-workers successfully prepared nano-dendritic Pt_3_Ni alloy nanocrystals with uniform size, good dispersion and high catalytic activity by one-step method [96]. Because of its simplicity, one-step method has promising application prospects, so it has become the focus of researchers’ work. However, the main shortcoming of this strategy is that there are still some deficiencies in the shape control, so the one-step preparation of nanocomposites still faces great challenges.

Seed-mediated growing is regarded as a Cz method used in nanoscale, because the conducting principle and essential conditions are almost the same. The method refers to the first synthesis of a nano-material, and then through chemical reaction on its surface or internal preparation of another or more nanomaterials, including seed method, template method and so on [97,98]. Zhang’s group used seed method to synthesize star-like Au/Pd double-metal nanocrystals with novel morphology and high catalytic activity [99]. Seed-mediated growing is the most popular synthesis method in fabrication of nanocatalysts, because of its advantage that the morphology of nanocomposites is highly controllable, and the number of composite components is not limited. However, this multi-step method is cumbersome and not conducive to large-scale production and application.

Impregnation method refers to dispersing two or two nanomaterials into solvents, and then bonding them together by electrostatic interaction between nanomaterials to obtain nanocomposites [100]. Forde successfully synthesized homogeneous Pd-based gold nanoparticles with excellent catalytic performance for organic reactions by chemical vapor immersion method. The method is simple and easy to manipulate the shape and structure of the product due to the pre-synthesis of nanoparticles [101]. However, because the electrostatic interaction force is relatively weak, there are some limitations in the practical application of this method. Only some nanomaterials with strong electrostatic interaction force can be compounded by this method, and sometimes the composite components are easy to be separated.

Post-treatment method refers to changing the composition of prepared nanomaterials through chemical reaction to obtain new nanocomposites, which are mostly used in the synthesis of hollow materials [102,103]. For example, Wang’s group obtained hollow PtPd core-shell nano-dendritic crystals by chemical selective etching [104].

### 4.3. Case Studies of Multimetallic Nanocrystals

Synthesis of multimetallic nanocrystals has been studied by numerous researchers and the most acknowledged scientists are Xia and Li. However, this part of the work is as successful as the synthesis of noble metal nanocrystals, and by varying the parameters, particles with various morphologies can be obtained in one reaction system. In this section, we will divide the multimetallic nanocrystals into several categories and provide some specific examples.

PtM nanocrystals: PtCu, PtCo, PtNi, and PtFe wormlike nanowires were synthesized via chemical reduction of [Pt(acac)_2_] and the corresponding [Cu(acac)_2_], [Co(acac)_2_], [Ni(acac)_2_], or [Fe(acac)_2_] salts simultaneously in a solution containing toluene and oleylamine. The growth mechanism of nanowires was discussed on the basis of the intermediate sample observation when the reaction parameters were partially tuned precisely. PtM nanoparticles generated at the initial reaction stage and oriented attached into nanowires [105]. Wu presented a controllable synthesis method via “top-down” route, in which concave PtNi alloys were fabricated through chemical-etching process with the help of coordination. Different etching priorities on specific sites contributed to the concave structure generation. The new method provides a chance for designing unique bimetallic morphologies and structures [106]. Cao reported a two-step approach for preparing atom-dispersed PtCu dual sites that was alloyed with palladium nanocrystals. Firstly, Pd ultrathin nanosheets were prepared using CO to reduce the salt and confine the surface. Then, aqueous solution containing CuCl_2_·2H_2_O was injected into Pd ultrathin nanosheets prepared in situ as above. In the end, the aqueous dispersion of Pt/Cu was transferred into a K_2_PtCl_4_ HCl solution, and ultrasonically processed for about 45 minutes to form PtCu dual sites that was alloyed with palladium nanocrystals [107]. Novel PtPd multimetallic nanocrystals, such as decahedral star and nanoplate with truncated triangular shape, were fabricated via co-reduction route. Reduction kinetics of PtPd alloy nanocrystals was manipulated and crystal structure was controlled by using different reductants including polyvinylidene chloride (PVP) and ethylene glycol (EG). PVP exhibited weak power of reducing and made great effects on the structure evolution of the twinned crystals, especially on the process when initial small particles were generated and aggregated. This work helps us understand the kinetically controlled mechanism in twinned nanocrystal generation and growth in co-reduction method [108].

PdM nanocrystals: PdCu tripods with high purity were prepared. The arms of the tripods grew in three directions of <211>, and {211} facets covered the side surfaces. Tripods originated from nanosheet seeds with defective or twinned planes. In typical procedure of tripods etching using hydrochloric acid as corrosive agent, the tripods were suspended in aqueous solution in the presence of PVP, and a certain amount of hydrochloric acid was introduced into the mixture and heated at the temperature of 90 °C and stirred using a magnetic stirrer [109]. Yolk-shell nanocage structures of Pd@M_x_Cu_1−x_ (M = Pd, Pt, or Au) were synthesized successfully via the reaction of galvanic replacement between Pd@Cu nanocubes and the HAuCl_4_, Na_2_PdCl_4_, or K_2_PtCl_4_ ethylene glycol solutions, respectively. Ag are most commonly used as sacrificial templates, and Cu can also react with various metallic compound under relatively mild condition because of its low reduction potential of Cu^2+^/Cu. Capping agent of HDA (Hexadecylamine) covered the planes {100} of Pd@Cu nanocubes and prevented them from dissolved, so the dissolution had to break sites in corner and proceed toward the inner parts [110]. Xie et al. also demonstrated the fabrication of PdRh nanocubes of core-frame frameworks and concave surfaces by limiting the excessive growth of Rh atoms at the seed angle and edge of cubic Pd nanocrystals. Etching strategy was applied to get rid of Pd cores selectively from the core-frame structure of PdRh concave nanocubes, producing a novel open structured Rh nanoframes. There are two important factors for etching selection: 1) generation of cubic core-frame structure; 2) different oxidative corrosion resistance between the two metals [111].

AuM nanocrystals: Chen reported a method to prepare monodisperse intermetallic CuAu nanocrystals by way of diffusing freshly generated Cu atoms into the prepared Au nanocrystals. Typically, Au nanoparticles, Cu(CH_3_COO)_2_, TOA, and OA were mixed together and heated for a period of time, and then cooled till room temperature, and well dispersed CuAu nanocrystals could be collected and then transferred into hexane or other nonpolar solvents for dispersion. Reaction could be described as Equation (14).

(14) $$3\mathrm{Au}+{\mathrm{Cu}}^{2+}+\mathrm{OA}/\mathrm{TOA}\to {\mathrm{Cu}}_{3}\mathrm{Au}\mathrm{or}\mathrm{CuAu}$$

Newly generated Cu atoms with high activity, are easy to diffuse into the lattice of existing Au nanoparticles, followed by self-ordering. This was probably the main formation process of the intermetallic nanocrystals, which might provide a new way of thinking that the solid-state reaction could occur in the solution, in which monodisperse nanocrystals can be produced with homogeneous diffusion in less time and consuming less thermal energy [112]. Li and his group also developed another method for the preparation of PdAu bimetallic nano-alloys, in which Pd and Au nanoparticles as well as PdAu nanoparticles, Pd(acac)_2_ and aqueous HAuCl_4_ were applied as precursors. Oleylamine was used as both solvent and surfactant. The two precursors were dissolved in oleylamine at 60 °C and reduced by borane-tert-butylamine at 80 °C [113]. Zeng synthesized nanocrystals of not only binary hybrid but also ternary hybrid with the control of heterogeneous nucleating and crystal growing onto nanoseeds with different shapes and compositions. The locations where Au nucleated and grew were feasible to be controlled accurately and selectively [114]. Gannan et al. demonstrated a seed-mediated method to adjust the shape of PdAu bimetallic nanocrystals, and a series of morphologies were achieved based on the reaction kinetics control by way of adjusting the concentration of reductant, the amount of precursor, the temperature, and the Pd nanocrystals employed as the seeds. The concentration of reactants, the temperature of reaction, and the Pd seed shape, played an important role in the structure of resultant PdAu nanocrystals [115]. Li et al. provided a size and shape-controlled production of Au@Pd core-shell nanocrystals via seed-mediated method. Experimental parameters such as capping agent type, reductant, concentration, and the adding style and rate of the reactants were examined. Reaction kinetics was manipulated and different nanostructures including nanocube, concave cube, nanodendrite, rectangular bar, octahedral and concave octahedral nanocrystal, were fabricated. Multishelled nanocrystals of three-layered Au@Pd@Au and four-layered Au@Pd@Au@Pd could also be synthesized by this strategy [116].

AgM nanocrystals: Zeng et al. reported that Ag could nucleated and grew on only one {100} face, or three, or six equivalent {100} ones of Pd seeds. When the process was precisely controlled through the addition rate and the reducing rate of the precursor AgNO_3_, AgPd alloy nanocrystals of the above three novel structures were probably synthesized. Hybrid dimers were produced when Ag grew on only one face of Pd seeds; eccentric nanobars were produced when Ag grew on three equivalent faces of Pd seeds; core-shell nanostructures were produced when Ag grew on all the faces of Pd seeds [117]. Nanorattles of M@Au/Ag (M = Pd, Au or Pt) could be fabricated by a general method within mainly three steps: generation of M@Ag structured nanocubes, ultrathin deposition of Au shells, and replacement for nanorattles. For example, nanorattles of Au@Au/Ag were prepared. The overall size was 15 nm, and the thickness of the coating was about 2.5 nm [118].

## 5. Attempt of Base Metal Nanocrystal Synthesis

### 5.1. Base Metal and Their Nanocrystals

Compared with noble metals, base metals are usually relatively cheap and inexpensive metals, which includes a large series of metals such as nickel, copper, cobalt, iron, aluminium, and so on. Base metals are usually easy to melt and purify, whereas noble metals including gold, platinum, and silver, are difficult to extract. Gold, platinum, palladium, and silver are often used as the preferred metal in different application areas for their excellent properties. However, with the rising prices of noble metals in recent years, the use of base metals such as copper, nickel, aluminium, zinc and tin instead of expensive precious metals is the inevitable trend in order to reduce costs. Taking the ORR (oxydoreduction reaction) electro-catalysts for example, PEMFC (Proton-Exchange-Membrane-Fuel-Cells) are clean and highly efficient with only water as the by-product, and could be applied to various fields such as automobile industry, unmanned aerial vehicles, etc. However, large amounts of platinum are required for the sluggish kinetics of ORR on the cathode to serve as electro-catalysts, which severely limits their commercialization. The US Department of Energy set a target of 0.125 mgPGM/cm for platinum group metals in 2017. Therefore, if one wants to get rid of the limitation of platinum resource scarcity, the platinum load of the whole vehicle must be controlled within 10 g. At present, discovery of low-PGM or non-PGM catalyst system is the main way to reduce platinum loading. Pt alloy have been presented in the above section for the low-PGM purpose, and the exploration of monometallic of base metal nanocrystals for non-PGM catalyst will be shown in this section [119].

The chemical and physical properties of base metal nanocrystals differ from those of their bulk counterparts. For example, Ni nanocrystals with high surface area exhibits excellent ferromagnetic properties [120]. Ni nanocrystals have important applications in catalysts, sensors, electronic and electrical devices, and biomolecular separation [121,122,123,124]. Ni nanoparticles prefer to crystallize into fcc structure naturally, and the hcp structure is meta-stable and difficult to achieve [125]. Hcp and fcc formations are anti-ferromagnetic and paramagnetic, and ferromagnetic and super paramagnetic, respectively. Bond distance of nickel fcc nanocrystals is 2.499 Å, while that of nickel hcp nanocrystals is 2.665 Å. The increases in bond distance from fcc nanocrystal to hcp nanocrystal contribute to the difference in magnetic properties [126,127]. Fe exhibits higher catalytic activity on {111} crystal plane in ammonia synthesis system, but {100} crystal plane in nitrite reduction system has higher catalytic activity [128,129].

Three kinds of copper nanocrystals with different sizes were prepared by thermal injection method using copper oxide as copper source, octadecene as solvent, oleic acid and oleamine as ligands. The catalytic degradation of nitrophenol was carried out, and results showed that copper nanoparticles exhibit higher catalytic activity and has the potential to take the place of noble metals as catalysts [130]. Liu et al. prepared copper nanomaterials by a green synthetic method. The catalytic ability of silver nanoparticles and gold nanoparticles was compared to objectively evaluate the activity of Cu catalysts. Results showed that copper metal nanocrystals have the strongest catalytic activity (Cu > Ag > Au) [5,131]. Cu nanostructures are of great importance for microelectronics application and catalysis application. For instance, Cu nanowires can be applied as the interconnects in electronic chips, and Cu nanoparticles can be applied in catalysis for various reactions such as gas detoxification or water-gas shift [132,133,134,135,136].

Both the fundamental scientific studies and practical industrial applications should be based on the availability of well-defined nanocrystals with controllable shape and size, as well as large scale production in good uniformity. However, synthesis of base metal nanocrystals such as Ni and Cu remains in the rudimentary development stage compared to the great success achieved in noble metals synthesis. On one hand, the standard electrochemical potentials of base metal are lower than that of precious metals, and the condition for base metal reduction is more critical. On the other hand, it is more difficult to reduce high-valence ions into low-valence metals in aqueous solution [137].

### 5.2. Synthesis Methods

Base metal such as nickel, copper and cobalt were commonly applied as components for synthesis of alloy nanocrystals together with precious metals. However, mono- base metal nanostructure was relatively less explored. The standard electrochemical potentials of base metal are lower than that of noble metals. Therefore, there are fewer reducing reagents for the production of base metals, and even when a certain reducing reagent can be applied for reduction of both base metal and noble metals, the condition for base metal reduction must be more critical compared to the noble metal reduction. Finally, the manipulation of the size and shape of base metal crystals becomes more complicated because there are fewer parameters that can be adjusted. Although lots of scientists have made great attempts for the synthesis of base metal nanocrystals, no results have been published that demonstrate that one can fabricate different perfect nanocrystals with uniform size and well dispersion as Pt, Pd, Au, and Ag. Herein, we can only summarize the preparation methods of ordinary powders, taking Ni as the typical example.

A series of synthesis methods have successfully developed to produce ultrafine Ni powders, including sonochemical synthesis, electrodeposition, electrochemical corrosion, and thermal decomposition and so on [138,139,140,141]. However, most of the methods seem not appropriate for mass production and shape control at the same time, in consideration of the complex of technique and the high cost of equipment. Chemical reduction route deserves simple technique and equipment that is suitable for both basic scientific research and large-scale production.

According to different classifications of reducing agents, liquid phase reduction methods include sodium hypophosphite reduction, sodium borohydride reduction, autoclave hydrogenation reduction, hydrazine hydrate reduction, polyol reduction and so on. The following is a review of the research progress of several methods.

Literature reports and our experimental results showed that using sodium hypophosphite or sodium borohydride as reductant, it is easy to obtain fine nickel powder with good sphericity [142]. However, the products obtained are Ni-P and Ni-B alloys rather than pure metal nickel powders. Their reaction processes are shown in Formulas (14) and (15) [143]:

(15) $${\mathrm{Ni}}^{2+}+4H_{2}{\mathrm{PO}}_{2}{}^{-}+H_{2}O\to \mathrm{Ni}+3H_{2}{\mathrm{PO}}_{3}{}^{-}+P+H^{+}+3/2H_{2}$$

(16) $$2{\mathrm{Ni}}^{2+}+2{\mathrm{BH}}_{4}{}^{-}+4H_{2}O\to 2\mathrm{Ni}+B{\left(\mathrm{OH}\right)}_{4}{}^{-}+B+3H^{+}+9/2H_{2}$$

Ni-P and Ni-B alloys are amorphous, and there is no directional growth. The primary particles are smaller nanoparticles. Therefore, it is easy to agglomerate into smooth spherical particles, and even coating can be formed when applied in electroless plating. However, the conductivity of amorphous alloys is poor, which is not suitable for the preparation of nickel powder for conductive pastes [144,145,146].

The oxidation product of hydrazine hydrate is nitrogen, the oxidation products of hydrogen is water, and the oxidation products of polyols are organic liquids or carbon dioxide and water, which do not pollute the products. Therefore, pure nickel powder can be obtained by using hydrazine hydrate, hydrogen and polyol as reducing agents. The reduction processes of hydrazine hydrate and hydrogen are as follows [147]:

(17) $${\mathrm{Ni}}^{2+}+2{\mathrm{OH}}^{-}+H_{2}\to \mathrm{Ni}+2H_{2}O$$

There are different opinions about the reduction process of polyols. Fievet et al. first proposed a two-step oxidation method [25]:

(18) $${\mathrm{Ni}}^{2+}+2{\mathrm{CH}}_{3}\mathrm{CHO}+2{\mathrm{OH}}^{-}\to \mathrm{Ni}+{\mathrm{CH}}_{3}\mathrm{CO}-{\mathrm{COCH}}_{3}+2H_{2}O$$

Zhou et al. proposed the mechanism of the complete oxidation of polyols to carbon dioxide and water based on their experimental results [148].

Spherical nickel powders are the most common morphology among the prepared fine nickel powders. In the preparation of micro-spherical nickel powder by hydrazine hydrate reduction method, Kim and other scientists have done a lot of work and made great progress. Fine nickel powder with good dispersibility has been prepared. The particle size is 0.27–0.85 um. However, the surface of nickel powder particles is burred and not smooth enough [147,149]. Park et al. prepared nickel powders by changing the order of adding reductant and strong alkali to realize reduction from nickel-hydrazine complex, which had a good effect on improving the surface structure of nickel powders [150].

Autoclave hydrogenation and reduction was thoroughly studied by Liang’s group from Institute of Process Engineering (formerly named Institute of Chemical Metallurgy), Chinese Academy of Sciences [151,152]. They used anthraquinone and palladium chloride as catalysts respectively to prepare ultrafine nickel powders by hydrothermal hydrogenation reduction. By adjusting the parameters of catalyst dosage, temperature, hydrogen partial pressure, nickel ion concentration and pH value, spherical nickel powders with controllable particle size was prepared.

Polyol method was also used to prepare micro-spherical nickel powders, and spherical-like nickel powders with high density were obtained. However, the cost of polyols as reducing agent is high, and the reaction temperature is high and the reaction time is long. Later, this method was improved by combining hydrazine hydrate reduction with hydrazine hydrate reduction to prepare nickel powder in polyol medium, and good results were obtained [153,154]. Others have studied the reduction of nickel powder by hydrazine hydrate in mixed media, and obtained better results than that in pure water media [155].

In the liquid phase reduction method, for the sake of controlling the particle size and dispersibility of nickel powders, the method of adding nucleating agent and surfactant is usually adopted. Shen et al. researched on the influence of adding PVP and AgNO_3_ on the size, shape and dispersion of nickel powder [156]. The results showed that these additives played a key role in preparing well dispersed submicron spherical nickel powders. In addition, the preparation of ultrafine nickel powders by liquid phase reduction could also be affected by changing the heating method. Kim et al. reduced nickel chloride with hydrazine hydrate in ethanol-water medium [157]. Spherical ultrafine nickel powders were prepared by traditional hydrothermal method and microwave hydrothermal method, respectively. The results showed that microwave hydrothermal method took less time, and the nickel powders prepared by microwave technology had more regular spherical shape, narrower particle size distribution and smoother surface. The obtained nickel powders met the application requirements of MLCC inner electrodes. In the liquid phase reduction method, for the sake of controlling the particle size and dispersibility of nickel powders, the method of adding nucleating agent and surfactant is usually adopted.

Generally, the crystal form of nickel powders is fcc, and some nickel powder with different hexagonal structure (hcp) has been prepared in liquid phase. The liquid phase method has the advantages of simple process, small industrial scale-up investment, small particle size and uniform distribution, and can also prevent the growth and agglomeration of nickel particles and improve the oxidation resistance by adding surfactants. However, XPS showed that Ni (OH)_2_ existed within surface of Ni powders prepared by liquid phase method. The surface of nickel powder prepared by liquid phase method was not smooth, and there were many holes or burrs. However, the liquid phase reduction method has incomparable advantages in particle morphology control, which will be introduced in the following section.

### 5.3. Case Studies of Base Metals

Shin et al. synthesized Ni nanoparticles of 5–12 nm on carbon via a thermal reduction. Typically, cellulose nanocrystal suspension and aqueous Ni(NO_3_)_2_ solution were put into a centrifuge tube, and mixed well by shaking. White-colored composite films obtained after centrifugation were air-dried at room temperature overnight and subsequently thermally processed at certain temperature for 2 h in a tube furnace under N_2_ [158]. Winnischofer et al. demonstrated a single-step synthetic method to produce Ni nanoparticles, the particle size distribution of which was narrow and the average size was about 416 nm, which exhibited a highly disordered atomic arrangement. The crystallinity could be improved by a post-synthesis annealing [159]. Peng et al. reported a single-step synthetic method for superparamagnetic colloidal nickel clusters. The formation of superparamagnetic nanoparticles was firstly produced in a high-temperatures polar media, and subsequently assembled to final nickel clusters. The morphology and corresponding magnetic properties of the superparamagnetic clusters can be manipulated feasibly by changing the concentration and temperature, respectively. The synthesized colloidal nanocrystal clusters exhibited capturing- and anti- bacteria ability for either Gram-positive or negative ones, and also bacterial spores [160]. Gao et al. reported a wet chemical method for Ni flower-like nanostructures produced under the magnetic field. In a typical procedure, NaOH solution, hydrazine solution, NiCl_2_·6H_2_O solution, and sodium hydroxide solution were prepared ahead of time. NiCl_2_·6H_2_O solution and hydrazine solution were together added into the NaOH solution at the temperature of 60 °C exposed in a magnetic field. NaOH solution was added to adjust the pH value till 12 at the beginning of the reaction. The solution turned black gradually after agitating for 20 min, and completely became black after another 25 min. The average particle size of the synthesized Ni nanostructures would decrease as the concentration of Ni^2+^ irons increased till 12 [161]. Li et al. reported a one-pot synthetic method for preparing nickel monodisperse nanoparticles, and the average size could be controlled at 4.8 nm, 6.4 nm and 11.3 nm. Typically, a mixture of nickel(II) acetylacetonate, 1,2-hexadecanediol and oleylamine was magnetically stirred at a temperature of 65 °C under the protection of high-purity Ar until the solid reactants was dissolved into solution completely. Then trioctylphosphine was injected into the flask, heated at 120 °C for 10 min and subsequently raised to 210 °C and heated at that temperature for another 45 min. TOP concentration, Ni(acac)_2_ concentration, and reaction temperature were key parameters that influence resulting particle size. High ratio of TOP/Ni(acac)_2_ and low temperature lead to small-sized Ni nanoparticles [162].

**Figure 8:**
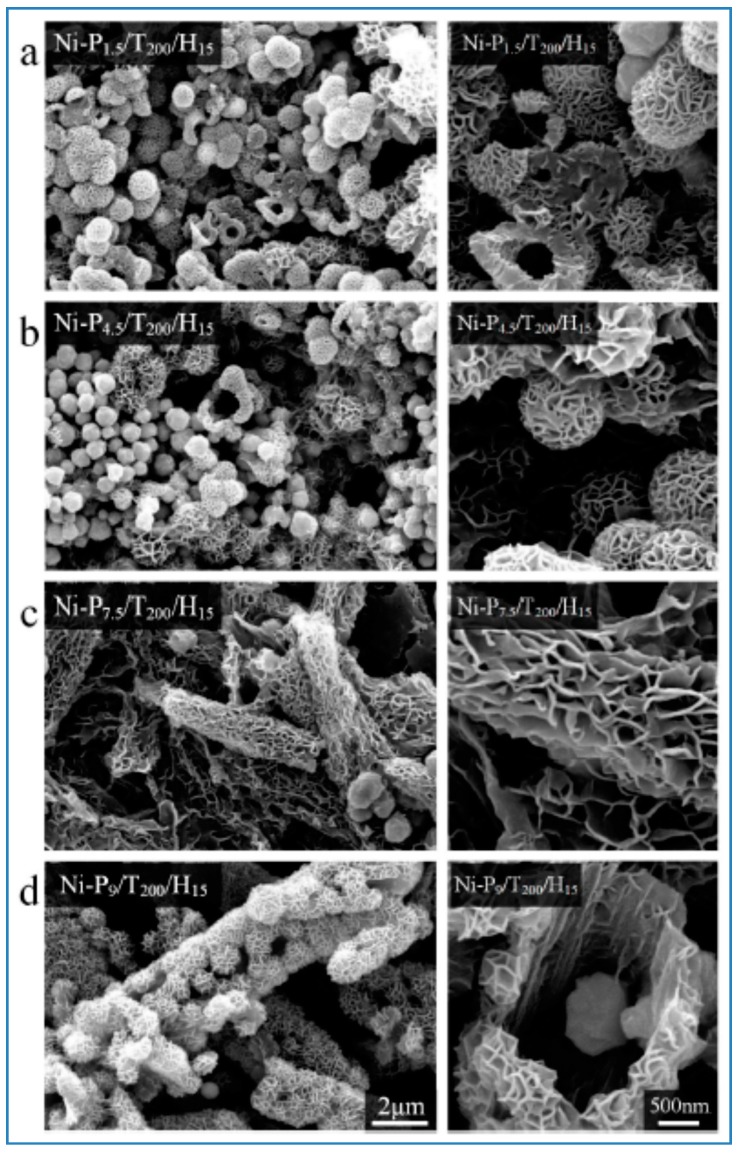
SEM images of Ni-P_1.5_/T_200_/H_15_ (**a**); Ni-P_4.5_/T_200_/H_15_ (**b**); Ni-P_7.5_/T_200_/H_15_ (**c**); and Ni-P_9_/T_200_/H_15_ (**d**) powders. The scale bars apply to all images in a column. Reproduced from [141] in Materials published by MDPI.

Pu et al. reported a one-step solvothermal method to fabricate submicron self-assemblies of Ni particles with no surfactant assistance. A series of structures such as flower-like, hydrangea-like, chain-like, sphere-like, and hollow-shaped architectures, could be synthesized. Parameters including reaction temperature, heating time, reactant type and concentration had great effect on the resulting products. In particular, PEG played an important part as a surfactant in the Ni particle generation with stoichiometry impacts. Growth mechanism for the formation of the structures was proposed based on the experimental results, and the as-synthesized Ni particles had good thermal stability. [141,163]. SEM images of self-assembled Ni particles are shown in Figure 8.

**Figure 9:**
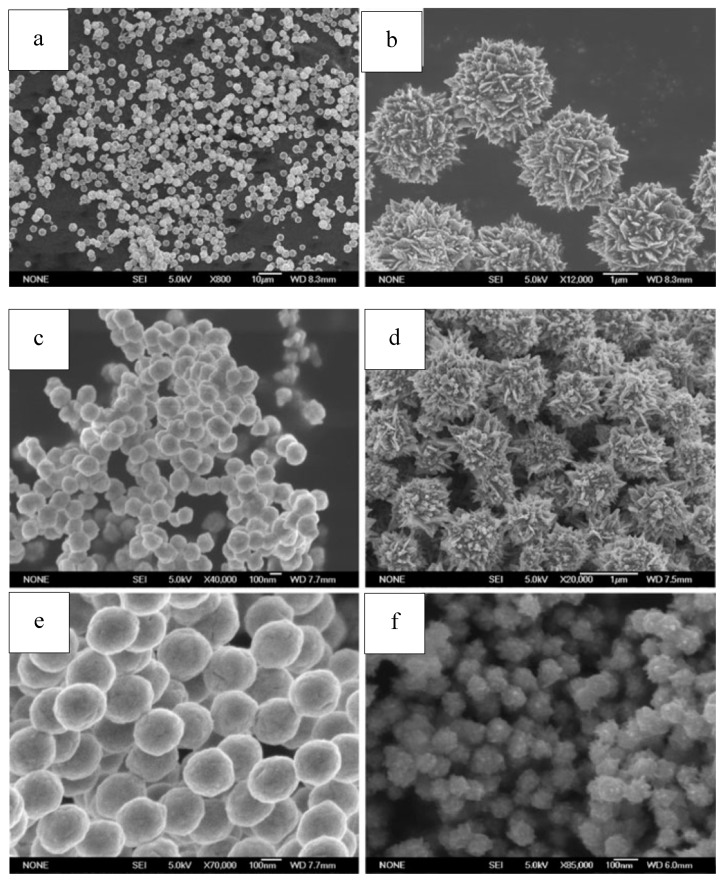
SEM of Ni particles from our work ((**a**,**b**). Ni obtained using hydrazine hydrate as reducing reagent, with no surfactant introduced; (**c**). Ni obtained when excessive NaOH (NaOH/Ni = 2.5) was used; (**d**). Ni obtained when the molar ratio of N_2_H_4_/Ni was raised to 6 and no NaOH was added; (**e**). Ni obtained when ethylene glycol was used; (**f**). Ni obtained when ethanol was used). Reproduced with permission [164]. Copyright 2012, Springer.

We have synthesized Ni powders with good uniformity and dispersion using N_2_H_4_·H_2_O as reducing reagent in the absence of any surfactants. The structure of the resulting particles varied from spheres with smooth surface to clusters made of nanosheets, when the applied solvent and alkali agents was changed and the ratio alkali/Ni was simply adjusted. FESEM (Field Emission Scanning Electron Microscope) images of the as-synthesized samples are shown in Figure 9. The generation of well-dispersed flower-like clusters might experience a two-step process: First, the generated Ni species aggregated together and form a solid core, subsequently active pot on the core surface grew into nanoplates and form the flower-structured architectures. The two-step mechanism of flower-like structure formation was attributed to a unique two-step successive reduction process that was adjusted via the pH value control [164,165,166].

**Figure 10:**
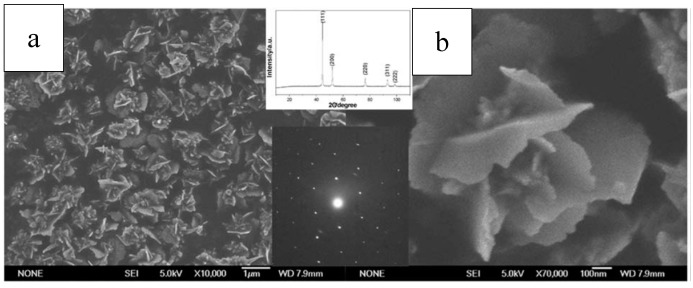

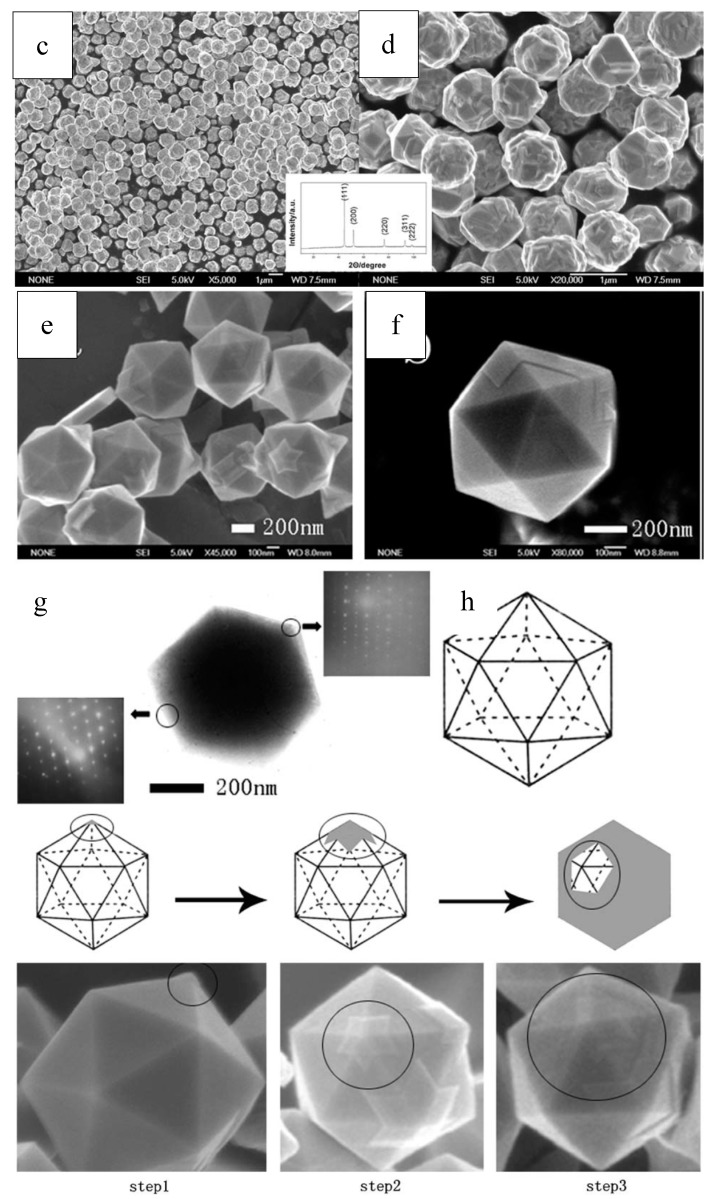
SEM and illustration of Ni particles synthesized via polyol method (**a**,**b**: Nanoflowers; **c**,**d**. quasi-spheres; **e**,**f**. icosahedra; **g**,**h**. Growing process of icosahedra). Reproduced with permission [167]. Copyright 2009, Elsevier.

We have also successfully synthesized nickel powders of various morphologies such as microspheres and icosahedra, as well as cluster architectures consisted of nanoflakes using the polyol method and demonstrated that the alkalinity of the solution had played an initial role during the generation of these unique nano- and micro- structures. The samples synthesized at a NaOH concentration of 0.4 M consisted of platonic icosahedra. The samples synthesized at the NaOH concentration above 0.5 M consisted of flower-like clusters. The sample synthesized when a mixture of H_2_O and EG was used as solvent, with a volume H_2_O/EG ratio of 0.25 and a NaOH concentration of 0.25 M, consisted of uniform quasi-spheres of 1 μm. Based on the famous Bravais’ rule and in consideration of the experimental observation, we proposed a new growth mechanism that icosahedra formed via a layer-by-layer process. The SEM and growth illustration were given in Figure 10 [167].

Tehrani et al. synthesized Ni nanocrystals with the diameters of no more than 10 nm via-fast scan-voltammetry method using Ni plating solution. The provided technique could produce non-equilibrium nanocrystals with hcp structure which indefinitely retains the stable phase. It was discovered that the size of the as-synthesized nanocrystals is mainly related to the interaction of nucleation and crystal growth rates. The present deposition process was a fast process during both nucleation and crystal growth stage. The graphite substrate played a key role during the kinetically-controlled electrochemical deposition process for stability of the hcp structured nickel nanocrystals. [127].

Zeng demonstrated a unique vapor liquid sectional flowing technology for continuous production of Ni nanoparticles in a microstructured reactor. NiCl_2_·6H_2_O was dissolved in anhydrous ethanol and reduced by N_2_H_4_·H_2_O. First, NiCl_2_·6H_2_O was dissolved in ethanol for NiCl_2_ solution, and NaOH was dissolved in hydrazine hydrate and ethanol mixture for hydrazine hydrate solution. Surfactant like CTAB or PVP were dissolved into NiCl_2_·6H_2_O solution when necessary. Both solutions were pumped into a caterpillar at a fixed rate and with a ratio of 1/1. The particle size was adjusted via optimizing experimental parameters including temperature, concentration, and the surfactant. Particles size decreased and its distribution became narrower when the reaction temperature increased and surfactant such as CTAB presented [168].

The above review mainly focuses on the typical synthesis of Ni particles. It can be seen that the manipulations of both size and morphology are not as successful as that during the noble metal synthesis. Similar conditions exist for other base metals such as Cu and Co nanocrystal synthesis. Cu nanocrystal synthesis is a bit better than other metals, but it is still far from satisfactory [137]. However, their bimetallic nanocrystals, along with the bimetals between noble and base metals mentioned in Section 4, may provide some new ideas.

**Figure 11:**
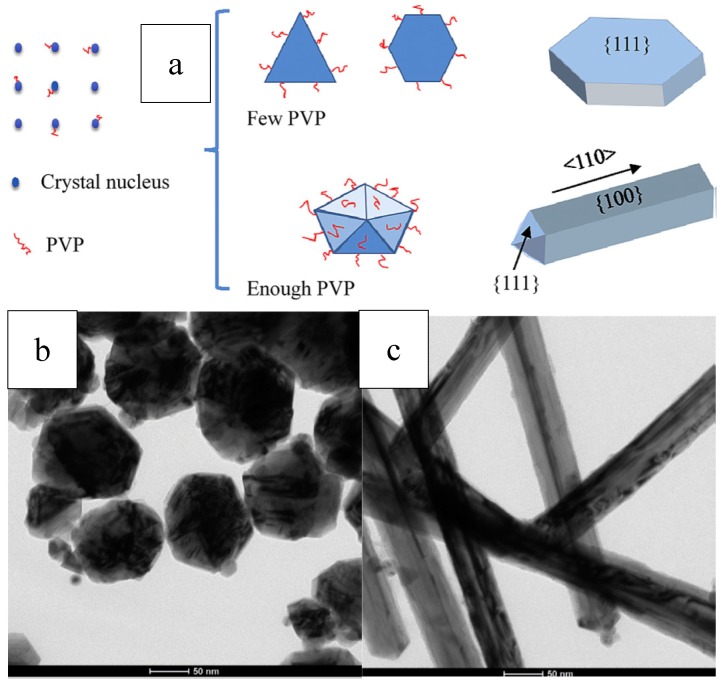
Schematic drawing of the formation (**a**) and SEM images (**b**,**c**). Reproduced with permission [169]. Copyright 2018, Elsevier.

For example, CuNi bimetallic nanocrystals of various morphologies and structures were successfully fabricated via solvothermal method. Cu(acac)_2_, Ni(acac)_2_, aniline, and surfactant PVP were used as starting materials and dissolved in benzyl alcohol, and vigorous stirred for 10 min, followed by sonicated for another 10 min. The mixture solution was then filled in the Teflon-lined autoclave. The vessel was sealed and heated at a specific temperature for 720 min. The product was collected and washed after cooled to the ambient temperature. The PVP amount could change the morphology of the obtained nanocrystals significantly. The reaction kinetics is tuned by varying the pyrrolidone unit concentration, leading to the transformation of CuNi structure to nanowires from hexagonal nanoplates. Temperature also played crucial role in controlling synthesis process of nanocrystals. With increase of reaction temperature, the morphology of nanocrystals diversified and the size of nanocrystals increased. It could be observed Ni atoms obviously inserted in the Cu lattice, generating fcc structured CuNi nanocrystals. The as-synthetic hexagonal CuNi nanoplates and CuNi nanowires were of good dispersion and uniformity, and high crystallinity and purity [169]. Figure 11 presents the schematic drawing of the formation (a) and SEM images (b-c).

## 6. Perspective Conclusions

An explosive growth in metal nanocrystal synthesis and research had been in progress for the last decade. One reason for this is that metal nanocrystals exhibit superior properties to their bulk counterparts and play an irreplaceable role in various applications such as catalysts, electronics, sensors, information storage, and so on. Another reason is that the synthesis of metal nanocrystals in large quantity and good uniformity can act as a unique unit to promote scientific research. For example, based on the successful fabrication of Ag nanocrystals with different exposed facet and similar size, catalytic activity of them can be compared, which will help for catalyst selection and in turn their preparation.

A large number of strategies have been designed and demonstrated for synthesis of metal nanocrystals, including plasma assisted chemical vapor deposition, electrodeposition, thermal decomposition, physical evaporation, and wet chemical reduction, among which, the wet chemical reduction method is most commonly used for the feasibility and simplicity of not only technology but also the synthesis equipment. The reactants of either metal precursor or reducing agent can be chosen from a variety of chemicals. The chemical composition, particle size, and structure shape of metal nanocrystals are easily adjusted by optimizing experimental parameters such as solvent and concentration, reacting temperature and time, capping agents and their amount. Various specific techniques have been demonstrated, including templating methods, seed-mediated growth, ligand control, oriented attachment, chemical etching, and Oswald ripening.

Scientists have made significant progress in the past decade due to the fact that noble metallic nanocrystals of a series of composition, sized, and shape can be fabricated and used in wide ranges of fields. Exquisite structures as various as we can imagine are synthesized, including nanospheres, nanorods, nanowires, nanobelts, nanocubs, icosahedra, decahedra, octahedra, tetrahedra, triangular plates, nanocages, nanoboxes, and so on. Compared with the overwhelming achievements in noble metals, synthesis of base metallic nanocrystals is far from satisfactory till now. The synthesized structures are usually limited within the aggregates and clusters. Rarely does research provide a universal method that can fabricate different base metallic nanocrystals by simply varying the experimental parameters. Therefore, it is worthwhile to make great efforts to make full use of the successful experience in noble metal nanocrystal synthesis and strengthen the research of base metal nanocrystals.

Although many groups have shown great interest in the fabrication of base metal nanocrystals, it is still comparatively poorly explored in most aspects as compared to the tremendous success achieved in noble metallic synthesis. One potential reason is that the standard electrochemical potentials of base metal are lower than that of precious metals, and the condition for base metal reduction is more critical. As a result, the manipulation of the nanocrystal size and structure of base metals becomes more complicated because there are fewer parameters that can be adjusted. Another potential reason is the complexity of base metal atoms and crystals themselves lead to the tendency to aggregate.

From the success of noble metallic nanocrystal synthesis, it can be concluded that thermodynamics related to precursor and reductant, kinetics within nucleation and crystal growth, and physical restrictions provided by the surfactant should must be comprehensively considered as ways to manipulate the component, shape, and size of nanocrystals. Therefore, breakthroughs in base metallic nanocrystal synthesis should also be based on the above three points. Specific methods include seed-mediated growth, ligand control, oriented attachment, chemical etching, and Oswald ripening, as mentioned in the main text. Furthermore, with the developing of in situ characterization technology, the reaction kinetics in the reducing process and crystal formation process will be observed directly and described more accurately. It is believed that the level of shape-controlled fabrication of nanocrystals realized in noble metals will also be achieved for base metals in the near future.

## Figures and Tables

**Table 1:** Reduction reactions with their corresponding E^0^ of common metals.

Metal Species	Reduction Reaction	E^0^(V)
Au	Au^3+^ + 3e^−^ = Au^0^	+1.50
Pt	Pt^2+^ + 2e^−^ = Pt^0^	+1.18
Ir	Ir^3+^ + 3e^−^ = Ir^0^	+1.16
Pd	Pd^2+^ + 2e^−^ = Pd^0^	+0.95
Ag	Ag^+^ + e^−^ = Ag^0^	+0.80
Rh	Rh^3+^ + 3e^−^ = Rh^0^	+0.76
Cu	Cu^2+^ + 2e^−^ = Cu^0^	+0.34
Ni	Ni^2+^ + 2e^−^ = Ni^0^	−0.25
Co	Co^2+^ + 2e^−^ = Co^0^	−0.28

**Table 2:** Oxidation reactions with their corresponding E^0^ of common reductants.

Reductant Species	Oxidation Reaction	E^0^(V)
H_2_	H_2_ = 2H^+^ + 2e^−^	0.000
N_2_H_4_·H_2_O (acidic)	N_2_H_5_^+^ = N_2_ + 5H^+^ + 4e^−^	0.230
N_2_H_4_·H_2_O (basic)	N_2_H_4_ + 4OH^−^ = N_2_ + 4H_2_O + 4e^−^	1.160
NaBH_4_	BH_4_^−^ + 3H_2_O = B(OH)_3_ + 7H^+^ +8e^−^	0.481
NaH_2_PO_2_	H_2_PO_2_^−^+H_2_O = H_2_PO_3_^−^ + 2H^+^ + 2e^−^	0.500
Na_3_C_6_H_5_O_7_·2H_2_O	C_6_H_5_O_7_^3−^ + 2H_2_O = 3CH_2_O + 3CO_2_ + 3H^+^ + 6e^−^	1.271
H_2_O_2_	H_2_O_2_ = O_2_ + 2H^+^ + 2e^−^	0.680
CH_3_OH	CH_3_OH = CH_2_O + 2H^+^ + 2e^−^	0.180
CH_3_CH_2_OH	CH_3_CH_2_OH = CH_3_CHO + 2H^+^ + 2e^−^	0.197
CH_3_CHO	CH_3_CHO + H_2_O = CH_3_COOH + 2H^+^ + 2e^−^	0.390
C_6_H_8_O_6_	C_6_H_8_O_6_ = C_6_H_6_O_6_ + 2H^+^ + 2e^−^	0.077
C_6_H_8_O_7_^2−^	C_6_H_8_O_7_^2−^ = CH_3_COCH_3_ + 3CO_2_ + 2H+ +2e^−^	1.100
